# Challenging Encounters: A Systematic Scoping Review to Understand Patients' Influence on GPs' Compliance With Imaging Guidelines

**DOI:** 10.1111/jep.70374

**Published:** 2026-02-18

**Authors:** Lizzie De Silva, Melissa Baysari, Peter Hanna, Jillian Clarke

**Affiliations:** ^1^ Discipline of Medical Imaging Science, Sydney School of Health Sciences, Faculty of Medicine and Health, Susan Wakil Health Building The University of Sydney, Camperdown Campus Camperdown New South Wales Australia; ^2^ Digital Health Human Factors Research Group, Susan Wakil School of Nursing and Midwifery The University of Sydney, Camperdown Campus Camperdown New South Wales Australia

## Abstract

**Rationale:**

The shift in healthcare from paternalistic to patient‐centred care has led patients to actively engage in their own health journeys. As gatekeepers, General practitioners (GPs) face growing pressure from patients who, having accessed online health information, request diagnostic imaging, often contrary to established clinical guidelines.

**Aim:**

This review explores the dynamic interactions among patients, GPs, and the broader healthcare system, offering insights into how to balance patient‐centred and value‐based care. The research specifically asked: what challenges do GPs face when responding to patient requests, and how do these challenges hinder adherence to guidelines on appropriate imaging? It also investigated what evidence‐based strategies GPs use to address patient requests and what support they receive in implementing guidelines and managing requests for imaging.

**Methods:**

The systematic scoping review was conducted according to the Joanna Briggs Institute's guidelines and reported in accordance with PRISMA‐ScR. Embase, Medline, Scopus, CINAHL, and Web of Science were searched for published peer‐reviewed articles.

**Results:**

Of 2137 potential papers, 11 studies met the inclusion criteria and revealed that patient expectations, anxiety, low health literacy, medico‐legal concerns, reliance on imaging capabilities, and institutional incentives for GPs often conflicted with evidence‐based imaging practices. GPs utilised strategies such as patient‐centred trust‐building, clinical expertise, and decision aids to address these challenges. A quality assessment of the papers revealed that most studies were of moderate quality, with Hoy's risk of bias tool used for quantitative papers and the CASP analysis for qualitative papers.

**Conclusion:**

This scoping review provided an understanding of the challenges encountered by GPs in addressing patient requests for imaging, underscoring the significance of patient‐centred care as being pivotal in nurturing a therapeutic patient‐GP rapport. Imaging is crucial for reducing diagnostic uncertainty and litigation; however, barriers such as economic incentives are present. Having interdisciplinary communication encourages guideline compliance. Implementing ongoing GP education, restructuring reimbursements and improving imaging accessibility can provide patients with a better understanding and purpose of the requested diagnostic imaging.

## 1 Introduction

Healthcare utilisation in Australia increased during the 2023–2024 financial year, driven primarily by an aging population [1], increasing chronic diseases [2], and the pandemic [3]. The pandemic also encouraged the adoption of digital health solutions such as telehealth and My Health Record [4]. For example, GP consultations for the aging population have risen from 70.8% to 96.6% in the 2023–2024‐year, consultations with medical specialists from 27.4% to 60.5%, and hospital emergency admissions from 7.2% to 26.7% [1]. Although it is uncertain whether this increase in the use of healthcare resources has benefited the health of society, some suggest that it has led to the overuse of medical services by healthcare professionals and patients [5]. One focus of growth has been in diagnostic imaging, with 25.6 million diagnostic services for 9.6 million Australians costing A$3.5 billion from 2013–2014 to 2018–2019 [6]. An overreliance on medical imaging for clinical diagnosis by general practitioners (GPs) and patients, shows one in five diagnostic services are of low value or unnecessary [7].

Digital apps [8], social media platforms [9], and widely advertised health awareness campaigns (S [10].)remind the public to be autonomous in their health management [11]. Online health information and government‐sponsored advertising can also contribute to patients feeling compelled to ask their GPs for diagnostic imaging tests, which could be seen as overservicing [10]. This places GPs in a position where their roles as ‘gatekeepers’ of patients' health and well‐being are relinquished towards more patient‐centric practices [12]. Tabenkin and Gross [13] provide a detailed description of gatekeeping:

…the authority to decide upon referrals to specialists, to implement the diagnostic work‐up and patient management in the primary care clinics, to consider finances when deciding about medical care, and to coordinate the actions of other caregivers, thus guaranteeing continuity of care.

[13, p. 75]

As responsible gatekeepers, GPs also need to inform patients of the risks versus the benefits of a test and balance the optimal against what the patient wants [14].

Patient factors play a crucial role in enabling best practices, with GPs reporting patient requests and preferences as one of the major impediments to GPs' clinical autonomy [15]. These impediments or barriers cause further dismantling of patient‐GP rapport, particularly when patients are dissatisfied with unfulfilled requests [16].

The long‐term consequences place a burden on the healthcare system, particularly in Australia, where the government funds a significant portion of medical imaging procedures with little or no cost to the patient [17]. Patients' desire for imaging may stem from concern about a particular disorder [18], a symptom [19], a recent diagnosis [12], or from non‐evidence‐based websites [16]. While patients have expectations of request fulfilment and GPs are pressured to oblige [15], there is little evidence about the challenges GPs face in addressing patients' requests for imaging and how they deal with them.

The overarching aim of this scoping review was to identify the challenges GPs face in addressing patient‐initiated requests for imaging studies and the strategies they employed around this dilemma, as described in the global academic literature. The research questions asked were:

*What challenges do GPs facein responding to patients' requests that are noncompliant with official imaging guidelines?*
*What strategies, using evidence‐based medicine, do GPs use to address patients'imaging requests? and,*
*What supports do GPs use to implement guidelines and manage patients' requests for imaging?*

## 2 Methods

This review was conducted according to the Joanna Briggs's Institute (JBI) guidance for scoping reviews [20] and is reported in accordance with the Preferred Reporting Items for Systematic Reviews and Meta‐Analyses (PRISMA) extension for Scoping Reviews (PRISMA‐ScR). The ‘participant, concept and context’ (PCC) approach to developing eligibility criteria was adopted. The ‘participants’ were GPs whose patients requested imaging; the ‘concept’ related to the challenges GPs encountered and the strategies employed to address patients' requests, and the ‘context’ was in primary care facilities [21].

A preliminary search for relevant published literature was conducted by the first author (LD), using Google Scholar, which determined there was a sufficient published literature base to proceed.A research librarian was then consulted to devise a search strategy. The databases searched were Embase, Medline, Scopus, CINAHL and Web of Science, from January 2010 to July 2023. This timeframe was chosen to keep the subject relevant to current standards of practice employed by GPs. Additional eligible articles not captured in the database search were identified through Google Scholar, and a search of the reference lists of included articles. The search terms were developed iteratively by a multidisciplinary team (see Supporting Information S1). The keywords were: patient request; preference; expectation and general practitioner; GP; doctor; physician with imaging; challenges and strategies.

The eligibility criteria for papers were those describing patients who requested diagnostic imaging from GPs, GPs who were recipients of such requests, and the challenges and strategies employed by GPs in addressing such requests. Selection criteria included studies reporting on: adult populations; patient‐initiated imaging; health practitioners' responses to patient requests for imaging; and those undertaken inprimary care facilities. Selections included all types of study design. Inclusion and exclusion criteria are listed in Table 1.

**TABLE 1:** Inclusion and exclusion criteria for study selection.

Title and abstract screening inclusion/exclusion criteria	
Inclusion	Exclusion
Adult population	Paediatric population
Studies reporting on patient‐initiated imaging	Health practitioner‐initiated imaging requests
Studies reporting on health practitioners' responses to patient requests/patient‐initiated imaging	Patients responding to health practitioners' directives
Primary care facilities only	Hospital setting or specialised/tertiary care setting
Secondary sources such as articles sourced from reference lists of included studies	Grey literature, reviews, and conference abstracts
Studies in English with full text available	No full text availability
All study designs	Any studies published before 2010
Studies written/translated into English	Non‐English studies

### 2.1 Data Extraction

Covidence (2023) *Covidence systematic review software* (Veritas Health Innovation, https://www.covidence.org) was used for data extraction, which included the study's title, year, author, country, aim, and design. Start and end dates, data collection methods, inclusion criteria, and participant numbers were also recorded. Additionally, elements related to the review were extracted, such as patients' reasons for referrals, practitioners' reasons for rejection, challenges, and reported interventions/strategies in the studies.

For this review, a broad operational definition for imaging requests by patients was established where the healthcare provider/GP was not acting on their own initiative to provide imaging services. Rather, they were influenced by patients' requests, expectations and/or demands.

The articles were initially imported into Endnote X9.3.3 (Clarivate Analytics, PA, USA) and then transferred to Covidence (Covidence systematic review software, Veritas Health Innovation, Melbourne, Australia). Reviewers (LD, JC and PH) independently screened the titles and abstracts of the first 10 articles in Covidence before discussions via Zoom meetings (Zoom Video Communications Inc., 2022) to ensure understanding of the process. LD conducted Title and Abstract screening for all articles initially, independently of JC and PH, who screened half each. Full‐text articles were then screened independently by LD and JC for eligibility. All conflicts were resolved through consultation and discussion. Details of the study selections and their characteristics are presented in Table 1.

### 2.2 Methodological Quality Assessment

Hoy et al. [22] risk of bias tool for quantitative studies [22] and Evans et al. [23] Critical Appraisal Skills Program (CASP) for qualitative studies were used to assess the quality of the included papers. Reviewers LD and JC, analysed the data from the quality assessment template from Covidence to evaluate the consistency and accuracy of the included studies [22]. For this review, a detailed critical appraisal of individual studies was performed. For the qualitative studies, articles were appraised on clear statements of:

- the aims (CASP 1),
- appropriateness of the research design (CASP 2‐3),
- recruitment strategy (CASP 4),
- data collection (CASP 5),
- bias (CASP 6),
- ethical issues (CASP 7),
- data analysis (CASP 8),
- research findings (CASP 9), and
- the value of the research (CASP 10) [23].

For the quantitative studies, ten questions were used to assess external validity (1–4) and internal validity (5–10). For our review, the number of ‘yes’ responses yielded a high risk of bias if the number of ‘yes's’ were 0–3, a moderate risk of bias if 4–7 'yes's', and a low risk of bias if there were 8–10 ‘yes’ responses [22].

By using both qualitative and quantitative appraisal tools, we presented our findings through narrative summaries rather than combining numerical scores. This approach highlights the methodological strengths and limitations of each study type. These appraisals helped us interpret the quality of evidence within each theme, aiming to reflect both the quantitative rigour and qualitative insights accurately.

### 2.3 Data Analysis and Presentation

**FIGURE 1:**
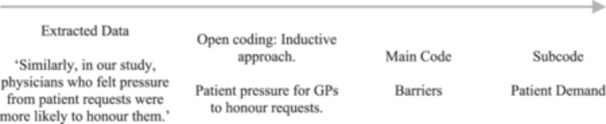
Coding sequence.

**FIGURE 2:**
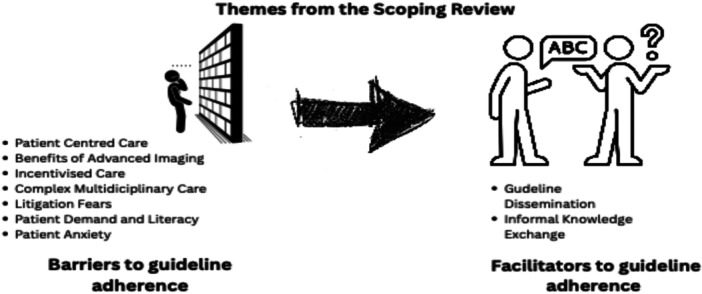
Emergent themes from the scoping review.

The data analysis included descriptive statistics for quantitative studies and theme‐based content analysis for qualitative ones. The research team used an inductive, collaborative process to develop coding from extracted statements, forming categories through NVivo (NVivo 14, released 2023). Articles were uploaded and open‐coded for relevant words or sentences related to research aims. Developed codes were grouped into categories with similar statements, which became themes aligned with the research objectives. A third reviewer (MB), not involved in screening or extraction, assessed coding and themes for an independent view. An example of the coding sequence is shown in Figure 1. Comparisons of themes discussed in the results below were explored to identify the overarching review objectives as indicated in Figure 2.

## 3 Results

### 3.1 Characteristics of the Included Articles

**FIGURE 3:**
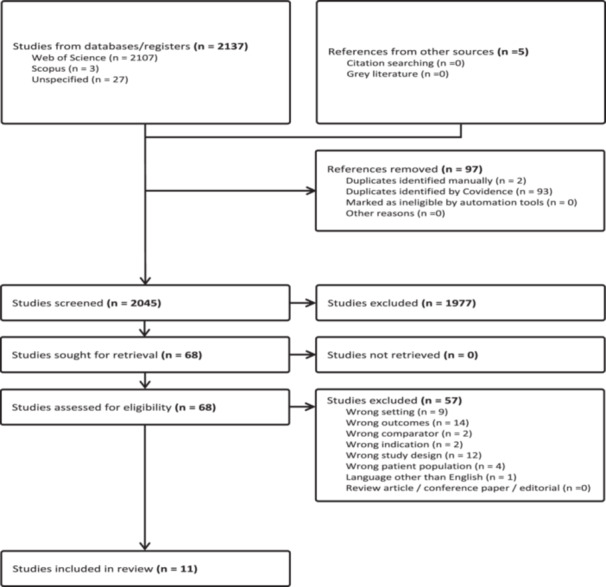
PRISMA flow diagram.

The search yielded 2137 studies with Covidence removing most of the duplicates (*n* = 93) and two removed manually. Five additional articles were retrieved from the Google Scholar search, resulting in 11 eligible articles, as shown in Figure 3.

Three papers were from the United States [24, 25, 26], two from Canada [15, 27], two from Norway [14, 28], and one each from the United Kingdom [29], the Netherlands [30], South Africa [31] and Australia [10]. The qualitative studies had between 10 and 30 participants, and the quantitative studies had between 60 and 360 participants. Table 2 summarises articles identifying barriers and facilitators.

**TABLE 2:** Characteristics of the articles included in the review.

Articles included	Year	Method	Sample size	Country	Imaging of focus	Aim	Challenges	Strategies
Botha & Mung'omba	2012	Survey	110	South Africa	X‐rays	To examine factorsthat may be associated with the practices of PCPs^a^ related toordering imaging in uncomplicated low back pain	Patient expectations Overcome embedded beliefs in the patients' perceived benefits of imaging. Poor patient health literacy	Decision aids Using decision aids in discussions with patients and building trust
Esfandiari, et al.	2019	Surveys	359	USA	General imaging	To elucidate physician‐perceived barriers to appropriatethyroid hormone management in clinical practice	Patient centred care GPs^a^ satisfying patients through honouring their requests. Pressured to comply with patients	Decision aids and valued clinical skills/trust Using decision aids in discussions with patients and building trust. Using past clinical experiences to be informed
Fenton et al.	2016	Randomised Controlled trial	61	USA	MRI	To assess the use of SPIs^a^ to enhance the patient‐centred communication skills of residentPCPs^a^ in the context of patient requests for low‐value diagnostictests	Patient centred care Patient satisfaction a priority for GPs through honouring their requests	Valued clinical skills/trust Maintaining therapeutic patient‐GP relationships
Gransjøen et al.	2018	Interviews	10–20	Norway	General imaging	To determine the perceived facilitators and barriers to guideline adherence	Benefits of imagingand litigation fears GPs perceived need to exclude diagnostic uncertainty through the use of imaging. GPs fear litigation should denied requests subsequently reveal serious missed pathologies	Guideline dissemination, valued clinical skills/trust and decision aids GPs keep themselves updated on available guidelines Using past clinical experiences to be informed Using decision aids in discussions with patients and building trust
Griffith et al.	2015	Interviews	12	Canada	General imaging	To gain a rich understanding of physician participants' experiences and processes of ordering DI^a^	Patient demand and literacy Poor patient literacy on health information	Valued clinical skills/trust Maintaining therapeutic patient‐GP relationships
Le et al.	2018	Surveys	168	USA	MRI	To identify factors that may be associated with the practices of GPs related to ordering imaging in uncomplicated low back pain	Benefits of imaging and litigation fears GPs' inexperience being a factor on the heavy reliance on imaging	Valued clinical skills/trust Using past clinical experiences Maintaining therapeutic patient‐GP relationships and building trust
Ottenheijm et al.	2014	Interviews	18	Netherlands	Ultrasound	To obtain a deeper understanding of GPs' perspectives on the diagnostic work‐up of patients withshoulder pain in general practice	Complex multidisciplinary care Disagreement between members within the interdisciplinary team	Valued clinical skills/trust Using past clinical experiences to be informed Less reliance on guidelines
Pike et al.	2022	Interviews	9	Canada	General imaging	To inform the barriers and facilitators using a theory‐based intervention (TDF^a^) in following imaging guidelines to reduce imaging for nonspecific (LBP^a^)	Benefits of imaging and litigation fears GPs fear of litigation should denied requests subsequently reveal serious missed pathologies	Valued clinical skills/trust Using past clinical experiences to be informed
Sajid et al.	2021	Clinical Record Audit	306	United Kingdom	MRI	The aim was to determine the appropriateness of use of MSK MRI in primary care	Complex multidisciplinary care Medicalising terminologies by interdisciplinary team members for normal age‐related problems Patient demand and literacy Misinterpretations of radiology reports by GPs	Informal knowledge exchange Interdisciplinary consultations
Sharma et al.	2021	Focus Groups	20	Australia	General imaging	To develop a public health campaign that was acceptable and understandable to community members and that had potential to reduce overuse of diagnostic imaging	Patient demand and literacy Patients' distrust in the health information given by health professionals	Valued clinical skills/trust, GP trust and guideline dissemination Shifting patients' perception about imaging (harms vs benefits). Promoting informed dialogues with patients Normalising ailment and providing alternative pathways. Using the waiting room for visual and emotional messaging for patients as educational tools
Walderhaug et al.	2022	Interviews	12	Norway	General imaging	To explore GPs' strategies in encounters with patients' expectations for imaging that are not medically indicated according to current guidelines, as well as how patients experienced these strategies	Patient anxiety Patient expectations due to anxiety, misinformation or prior experiences. Litigation fears GPs fear of misdiagnosis or clinical uncertainty Limited consultation time Pressure to preserve patient‐GP rapport	Valued clinical skills Using past clinical experiences to be informed Normalising ailments Deferring to professional authority

a Primary Care Practitioners (PCPs), Standard Patient Instructors (SPIs), Magnetic Resonance Imaging (MRI), General Practitioners (GPs), United States of America (USA), Theoretical Domains Framework (TDF), Low back pain (LBP), Musculoskeletal (MSK), Diagnostic Imaging (DI).

## 4 Results

### 4.1 Barriers and Facilitators to Guideline Compliance

GPs experienced several challenges in considering patients' requests which we categorised as barriers to guideline compliance. We also categorised facilitators that allowed GPs to comply with guidelines.

#### 4.1.1 Patient‐Centred Care

Four studies addressed patient‐centred care [24, 26, 27, 30]. A consistent theme that emerged was GPs' commitment to maintaining a therapeutic relationship with their patients by addressing their fears and anxieties. Legitimising patients' thought processes and adopting an agreeable approach led to greater patient satisfaction. A GP views the fulfilment of a patient's expectations as a therapeutic approach that enhances patient's acceptance of treatment outcomes. This acceptance often leads to improved adherence to GPs' recommendations, as patients feel empowered in having contributed to their own solutions [30]. Furthermore, Pike et al. [27] studyunderscored GPs' emphasis on prioritising patients' wellbeing over resource stewardship [27]. In that study, GPs were more concerned about appeasing patients' anxieties by ordering scans, even those which were not medically indicated, simply to pursue patient satisfaction. This was supported by participants in other studies investigating patient‐centred care with illustrative quotes from such studies.

Our finding shows that those ordering imaging were more likely to cite patient‐centred care as the rationale for the decision.

[26, p. 760]

GPs are increasingly facing pressure to enhance patient satisfaction in the context of time‐constrained visits. However, it remains unclear about how large an impact patient requests have on physician‐prescribing decisions and how they may be modified by perceived and actual patient satisfaction.

[24, p. 1542]

#### 4.1.2 Benefits of Advanced Imaging

GPs also believe that advanced imaging serves as a useful tool in diagnosis, particularly in primary care. Imaging is quicker, often painless, non‐invasive and can resolve diagnostic uncertainty [32]. A participant in Gransjøen et al. (2018) study explained the importance of imaging:

'For [us] GPs, this meant a shift in modality… Where you could only use plain radiography before, you now use MRI because you have gotten used to it'. This shows that GPs have grown accustomed to referring to modalities heavier on both information and use of resources and that they are viewed as highly useful (Gransjøen et al., 2018, p. 5).

#### 4.1.3 Incentivised Care

Several papers reported a range of factors that impacted GPs' compliance with guidelines with GP participants in Gransjoen's study (2018), indicating that working in private facilities and offering imaging services meant there was a greater demand for profits (Gransjøen et al., 2018). If scans were ordered, it would be favourable to the organisation and a way of incentivising GPs. Three studies, Pike et al. [27], Gransjoen et al. (2018) and Ottenheijm [30], mentioned the economic benefits offered by various health institutions for ordering more imaging. According to one radiologist in Gransjoen's study, the institution would offer incentives for increased numbers of tests performed, as it was profitable for the organisations offering the services. As stated by a participant in Gransjoen's study:

So, … many institutions who want customers right, I think an essential point here, [is] the privatisation of health care and running a health business rather than healthcare. That's scary, because … profit decides what you do…

(Gransjøen et al., 2018, p. 5)

Similarly, found that economic gain and incentivised GPs meant that patients had an expectation that all GPs working within the same system would accede to their requests. A participant in their study is quoted below,

Physicians reported that patients sometimes pressure them for images because other doctors at their place of work image more liberally and patients believe that to be a higher standard of care

[30, p. 240]

Moreover, with incentives becoming a driving factor, GPs were also seeing more patients, which pressured them to consult in a shorter timeframe [27]. Therefore, GPs argued that responding negatively to patients who seek imaging studies would take too long to explain, and it was easier to accede to patients' requests. In addition, in Pike et al. [27] study:

Participants [GPs] reported that it takes much longer to explain why an image is not needed than to simply order an image. They don't feel they have adequate time to convince patients that they don't require imaging in the run of a busy clinic day.

[27, p. 22]

#### 4.1.4 Complex Multidisciplinary Care

Esfandiari et al. [24] call this ‘fragmentation of care’, referring to noncontinuity of care due to differences in opinions on existing guidelines between the various disciplines or multidisciplinary healthcare systems. In an era of complex healthcare and multiple providers, there are differences in thinking among interdisciplinary team members. Such differences result in miscommunication within medical disciplines, resulting in guideline noncompliance, as stated by participants in Ottenheijm's [30] and Esfandiari's [24] studies respectively.

Orthopaedic surgeons think quite differently in the diagnostic work‐up compared to what is stated in the GP guideline. A number of patients also see the physiotherapists, [and] I think it is quite hard to communicate in such vague terms [using general guidelines for imaging] …

[30, p. 241]

Patients' requests for tests and treatments are commonly reported as barriers to appropriate management of thyroid hormones, in addition to patient nonadherence and multiple providers managing thyroid hormone therapy…

[24, p. 1539]

#### 4.1.5 Litigation Fears

Partially contributing to GPs fulfilling patients' needs was the fear of potentially missing serious pathologies even when symptoms were not ‘red flagged’ or considered critical under established imaging guidelines [27]. In cases where there was diagnostic uncertainty and patients were anxious, GPs relied heavily on imaging [28]. Furthermore, asa participant in Gransjøen et al's. (2018) study explains,

When someone comes in with a long‐lasting cough so you auscultate and think it may be a pneumonia, before you would think it probably is pneumonia. But now, for safety's sake, we take an x‐ray to make sure the diagnosis is correct.

(Gransjøen et al., 2018, p. 4)

Part of excluding uncertainty and avoiding litigation, emphasised by the majority of GPs in Gransjøen et al. (2018) study, was documenting all treatment plans so that it is on record for further reference (Gransjøen et al., 2018).

#### 4.1.6 Patient Demand and Literacy

Participants in Griffith et al. [15] study described how patients requested diagnostic imaging without a nuanced understanding of the optimal timing for detection, that is, when it was too early to detect an ailment by imaging or imaging more frequently than clinically recommended. This study stated in a quote on patient demands:

For example, if there was a plan to follow‐up on some finding in 6 months, the patient request might be 'why can't it be done next week, or in 3 weeks?' and usually that involves an explanation on what's being followed; if it's going to change, it's not going to change fast enough to make this worthwhile in which case, you've just had the radiation exposure without any realistic chance of it showing anything new.

[15, p. 22]

Griffith et al. [15] study also revealed that the majority of GPs were concerned about poor‐quality online content being sought by patients. They said:

Overall, the majority of participants expressed how patients may request DI [diagnostic imaging] because they have received misinformation or incomplete information and have not appraised the quality of that information.

[15, p. 22]

A South African study by Botha and Mung'omba [31] focused on the problem of unwarranted demand for radiological imaging by patients and how sociodemographic factors influenced their demands. Their study revealed that only a few patients who demanded images had a good understanding of the requested exam. In fact, only

10.9% demonstrated good knowledge about X‐rays. Those with average knowledge accounted for 15.5% of the study sample. The majority (73.6%) had a score of 0 or 1 and, as such were considered to having poor knowledge about x‐rays.

[31, p. 18]

#### 4.1.7 Patient Anxiety

One recurring theme from the included papers was that patients were anxious, particularly when they were misinformed about the merits and limitations of imaging studies. GPs in Griffith et al. [15] study responded to requests for imaging solely to ease patients' anxiety. One GP, quoted from their study, said:

Sometimes, if there's a significant amount of patient anxiety, or yeah, if they are not going to rest until that happens, then, I think, sometimes probably imaging is done in that case, unnecessarily.

[15, p. 22]

Moreover, anxiety can be further heightened by medicalising language—that is, assigning labels to everyday experiences (such as those commonly associated with aging) through an expanded disease definition [33]. These are included in radiology reports, prompting both GPs and patients to seek further testing.

These findings emphasise the need to equip primary care providers with strategies to manage patient anxiety through training and system‐level support, in order topromote adherence to imaging guidelines and reduce unnecessary diagnostic requests driven by reassurance‐seeking behaviour.

### 4.2 Facilitators to GPs Complying With Guidelines

Two themes emerged as facilitators of guideline compliance. Most GPs agreed that ease of accessibility and readability in a busy clinical setting enabled GPs to consult guidelines more efficiently.

#### 4.2.1 Guideline Dissemination

In their trial, Fenton et al. [25] assessed an educational intervention's effectiveness in raising resident GPs' confidence in managing unnecessary patient requests. The study showed no impact; however, Gransjøen et al. (2018) and Sharma et al. [10] found GPs and patients preferred printed educational materials, like booklets with imaging guidelines, over formal presentations by professional bodies. Gransjøen et al. (2018) quoted one GP on booklets' benefits:

Easy access was an important facilitator, since there usually was little time to look up guidelines as soon as they were needed.

(Gransjøen et al., 2018, p. 6)

According to the findings of Sharma et al. [10] study as quoted:

Participants [patients] felt the combination of the leaflet and poster messages would give them more confidence to ask questions. Specifically, the ‘Ask your doctor – do I need this test?’ message received almost universal praise across both groups [two focus group patients]—participants [patients] supported components of the campaign that empowered patients with information.

[10, p. 654]

#### 4.2.2 Informal Knowledge Exchange

Several GPs in Ottenheijm et al. [30] study noted that while generic guidelines often present challenges due to patients' varying needs, their practical application and ease of use encourage adherence. Additionally, radiologists in Gransjøen et al. (2018) study preferred informal knowledge exchange over formal national guidelines. They quoted:

…radiologists preferred more local protocols and informal knowledge exchange to formal, national guidelines. Local protocols and informal knowledge sharing were perceived as more up to date and therefore better suited to radiological departments.

(Gransjøen et al., 2018, p. 4)

With communication between colleagues within interdisciplinary teams, GPs referred patients demanding imaging to other members of the interdisciplinary team or encouraged patients to refer to guidelines by professional authorities. A summary of Barriers and Facilitators is presented in Table 3.

**TABLE 3:** Barriers and facilitators to guideline compliance.

Barriers	Articles investigating barriers
Patient centred care	[24, 26, 27, 30]
Benefits of advanced imaging	[28]
Incentivised care	[27, 28, 30]
Complex multidiscipline care	[28, 30]
Litigation	[27, 28, 30]
Patient demand and literacy	[15, 31]
Patient anxiety	[15, 29]
Facilitators	Articles investigating facilitators
Guideline dissemination	[10, 24, 25, 26, 27, 28]
Informal knowledge exchange	[14, 28, 30]

### 4.3 Strategies Employed by GPs to Reduce Unwanted Patient Requests

Several methods were employed by GPs in cases of either fulfilment or rejection of patient requests.

#### 4.3.1 Trust in the GP

Participant GPs in Ottenheijm et al. [30] study believed trust could reduce patient imaging requests. They emphasised the need for thorough clinical examinations to validate symptoms during GPs consultations. This sentiment was reflected in a quote from the study, showing confidence in GPs:

Yes, one advantage is that you extensively examine your patients. (…) Gain trust! And often a correct diagnosis, with subsequently less need for additional tests. Maybe these are ordered too much.

[30, p. 241]

Normalising ailments was often evident in long‐term relationships where GPs were familiar with their patients and did not appear overly concerned about the presented symptoms during clinical visits. Even when patients' requests were declined, patients expressed how GPs took them seriously and that they were confident in their GPs' final decisions. Such a positive response was related to the length or extent of the relationship between patients and GPs [14].

### 4.4 Valued Clinical Skills

Part of a thorough clinical examination includes patients' beliefs that good clinical skills (e.g., symptom recognition and knowledge of history) are essential at each visit. Experience with symptom recognition helps GPs normalise patient concerns and reassures them to adopt a ‘watch and wait’ approach before fulfilling requests. Pike et al. (2018) study discussed GPs' views on the benefits of good clinical assessments as follows:

Most reported considering the patient's history and physical exam findings when deciding whether or not an image is warranted. Other considerations include assessments for red flag conditions and surgical candidacy as well as response to previous treatments and resource stewardship.

[27, p. 12]

### 4.5 Decision Aids

Moreover, in addition to the ‘watch and wait’ strategy, GPs in Fenton's [25] study had built‐in software that aided in requesting appropriate tests and interventions based on their input about the patient. Such an algorithmic approach seamlessly allowed patients and GPs to come to a shared decision based on the algorithmic output [25]. Decision aids using evidence‐based medical software were used as interventions to address patient requests. However, the study by Fenton et al. [25] that explored the effectiveness of using patient‐centred techniques within decision aids revealed that they were not effective within the clinical setting. Fenton et al. [25] study noted that:

In the 155 encounters with [individuals posing as patients for the study] who requested low value tests, residents ordered tests in 41 visits (26.5% [95% CI, 19.7%–34.1%]). After adjustment for visit number and case, receipt of the intervention was not associated with a significant difference in the odds of requested test ordering (adjusted odds ratio, 1.07 [95% CI, 0.49–2.32]).

[25, p. 195]

**FIGURE 4:**
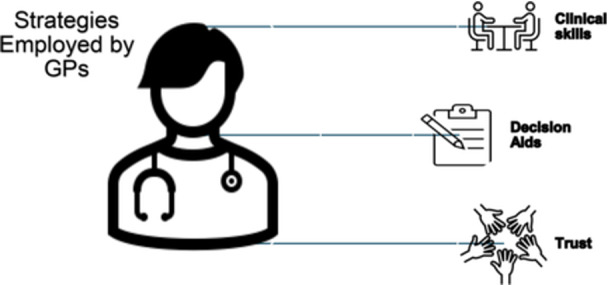
Strategies employed by GPs to address patient requests for imaging.

GPs thought it was difficult to use decision aids when patients were anxious or demanding, whereas implementing good clinical skills within long‐term GP‐patient interactions was more effective than relying on software. Figure 4 shows an overview of the strategies employed by GPs, as reported in the selected articles.

### 4.6 Methodological Assessment

Overall, most studies were moderate to high quality as per Hoy et al. [22] risk of bias tool [22] and CASP [34]. Two quantitative studies; Sajid et al. [29] and Fenton et al. [25], had a low bias risk, scoring 8 out of 10, while Botha and Mung'omba's [31] study had a high risk with a score of 4. Four of the six qualitative studies met all criteria, except Griffith et al. [15] study, which failed criteria 2 and 6, and Ottenheijm et al. [30], which did not meet criteria 4 and 6. Further, the review noted the potential bias risk by addressing appropriate methodology, recruitment strategy, and researcher bias (A detailed outline is available in the supporting information).

## 5 Discussion

This scoping review highlights the complexities GPs face when managing diagnostic imaging requests and the strategies they employ to navigate these challenges. Central to their approach is the cultivation of trust within the patient‐GP relationship. As GPs gain clinical experience and confidence, they are more adept at reassuring patients without always acceding to imaging requests. Techniques such as the ‘watch and wait’ approach and normalisation of symptoms are commonly employed to reduce patient anxiety and support shared decision making.

Despite an awareness of imaging guidelines, GPs often encountered difficulties in adhering to them. Our synthesis identified that the challenges GPs face in responding to patients' requests that are noncompliant with official imaging guidelines include patient expectations, time constraints, diagnostic uncertainty, and medico‐legal concerns. Guidelines were typically discipline‐specific, and for GPs to act as gatekeepers, they must be familiar with each discipline's guidelines while maintaining a patient‐centred approach. Such familiarity means GPs must take the initiative to become aware of the various guidelines, particularly those updated regularly. Pike et al. (2018) noted that GPs often prioritised patient satisfaction over resource stewardship, particularly in the context of diagnostic uncertainty and litigation risk.

The rise of online health information has further complicated consultations. Many patients access low‐quality sources, contributing to heightened anxiety and inflated perceptions of illness severity [33]. This, in turn, can pressure GPs to order imaging to reassure both themselves and their patients, while some GPs reported that misinformed patients increased the likelihood of unnecessary imaging.

Our review uncovered that the strategies GPs employ, based on evidence‐based medicine, to handle patients' imaging requests, were interdisciplinary collaboration and the presence of clear, accessible, and cross‐disciplinary resource guidelines. Radiologists and GPs noted that generic, consistent guidelines supported better compliance and improved clarity in decision‐making.

The review highlighted challenges of time‐pressured consultations, potential missed pathologies, and litigation fears. In a systematic review and meta‐synthesis of qualitative studies on misconceptions and evidence‐based medicine for low back pain, Slade et al. (2016) found that GPs reported perceiving the risk of missing underlying spinal pathology without imaging when managing uncomplicated back pain. Hall et al. [35] noted in their systematic review that most interventions aimed at reducing unnecessary imaging were not guided by behavioural changes of GPs' ordering practices and often failed to target known barriers such as patient pressure, difficulty explaining why imaging was unnecessary and lack of time.

Considering the broader implications for practice and policy around patient‐centred care and resource stewardship, involves supporting GPs to implement guidelines and manage patients' imaging requests. Achieving this requires systemic changes, including increasing imaging access, adjusting financial incentives for GPs, and enabling longer consultations. Our review demonstrated that an integral component of GPs reducing unnecessary costs was earning patients' trust through a strong therapeutic relationship. Croker et al. [36] found that patients' confidence was closely linked to their perception of being taken seriously by their GP. Trust building is crucial. GPs who perform thorough examinations and validate concerns reduce anxiety and imaging demand [14, 36]. Although denying requests may cause dissatisfaction (Jerant et al., 2017), many patients within a trusting relationship accepted denials when addressed respectfully (Ottenheihm et al., 2014).

Education and guideline format also play a role. Fenton et al. [25] found educational materials ineffective for trainees, whereas Gransjoen et al. (2018) noted experienced GPs preferred concise booklet‐style guidelines. However, applicability varies based on patient context, such as their psychological state (anxiety) or sense of urgency. Radiologists favoured informal exchanges with interdisciplinary team members. However, guidelines that conflict with traditional practice or run against the norm often encounter resistance from medical professionals who consider themselves well‐versed or experts in their specialty. This is particularly so in highly specialised areas of clinical work where certain practices have been strongly embedded over time [37].

## 6 Conclusion

This scoping review examines the challenges GPs face and the facilitators that help when patients request imaging studies. It also reviewed the strategies employed to address these needs. It highlights the significance of patient‐centred care in fostering therapeutic GP‐patient relationships by acknowledging patient concerns, and the crucial role of imaging for reducing diagnostic uncertainty and avoiding litigation. Moreover, health professionals were offered economic incentives that encouraged increased service usage, creating barriers toresource stewardship and guideline adherence. Effective interdisciplinary communication enhances guideline compliance and facilitates informal knowledge transfer among team members, keeping them up to datewith clinical guidelines. Implementing measures for GPs and patients can establish a healthcare system focused on patient care and guideline adherence. Proposed measures include enhancing imaging accessibility, revising reimbursement frameworks, and promoting evidence‐based medicine through continuous GP education and training.

## Conflicts of Interest

The authors declare no conflicts of interest.

## Supporting information


**Table 4:** Qualitative Assessment using CASP analysis. **Table 5:** Quantitative Assessment using Hoy et al's Risk of Bias tool.

## Data Availability

The data that support the findings of this study are available from the corresponding author upon reasonable request.
