# Supplementary material for: Challenging Encounters: A Systematic Scoping Review to Understand Patients' Influence on GPs' Compliance With Imaging Guidelines
Source: J Eval Clin Pract. 2026 Feb 18;32(1):e70374. doi: 10.1111/jep.70374 (PMC12917351; doi:10.1111/jep.70374)
Supplement: Supplementary file 1 — Table 4: Qualitative Assessment using CASP analysis. Table 5: Quantitative Assessment using Hoy et al's Risk of Bias tool. [file JEP-32-0-s001.docx]

**Supporting Information**

| **Search Strategy** |
| --- |
| 1. Patient*.mp 2. Request*.mp 3. Need*.mp 4. Desir*.mp 5. Prefer*.mp 6. Demand.mp 7. Expectation*.mp 8. Consumer*.mp 9. 1 and 2 or 3 or 4 or 5 or 6 or 7 10. Diagnostic imaging.mp 11. Radiolog*.mp 12. Imag*.mp 13. Xray*.mp 14. Computer tomograpy.mp 15. Ultrasound*.mp 16. Positron emission tomography.mp 17. Magnetic resonance imaging.mp 18. 9 and 10 or 11 or 12 or 13 or 14 or 15 or 16 19. 9 and 18 20. General practitioner.mp 21. GP*.mp 22. Healthcare provider*.mp 23. Primary car*.mp 24. Healthpractitioner*.mp 25. Doctor*.mp 26. Physician*.mp 27. 19 and 20 or 21 or 22 or 23 or 24 or 25 28. Strateg* or method* or mean*or process* 29. Guideline* or outline*or definition* 30. 28 and 29 31. 27 and 30 32. Limit 26 to yr.=’2010-current’ 33. Remove duplicates from 27. 34. Limit 28 to English language 35. Limit 29 to humans |

Table 4: Qualitative Assessment using CASP analysis

|  | No |  | Yes |  | Not Applicable |
| --- | --- | --- | --- | --- | --- |

| Author/Year | CASP 1 | CASP 2 | CASP 3 | CASP 4 | CASP 5 | CASP 6 | CASP 7 | CASP 8 | CASP 9 | CASP 10 |
| --- | --- | --- | --- | --- | --- | --- | --- | --- | --- | --- |
| Ottenheijm et al., 2014 |  |  |  |  |  |  |  |  |  |  |
| Griffith et al.,  2015 |  |  |  |  |  |  |  |  |  |  |
| Gransjoen et al,  2018 |  |  |  |  |  |  |  |  |  |  |
| Sharma et al.,  2021 |  |  |  |  |  |  |  |  |  |  |
| Pike et al.,  2022 |  |  |  |  |  |  |  |  |  |  |
| Walderhaug et al.,  2022 |  |  |  |  |  |  |  |  |  |  |

CASP tool for qualitative studies. CASP 1: Was there a clear statement of the aims of the research , CASP 2: Is a qualitative methodology appropriate? CASP 3: Was the research design appropriate to address the aims of the research? CASP 4: Was the recruitment strategy appropriate to the aims of the research? CASP 5: Was the data collected in a way that addressed the research issue? CASP 6: Has the relationship between researcher and participants been adequately considered? CASP 7: Have ethical issues been taken into consideration? CASP 8: Was the data analysis sufficiently rigorous? CASP 9: Is there a clear statement of findings? CASP 10: How valuable is the research?

Table 5: Quantitative Assessment using Hoy et al’s Risk of Bias tool

|  | No |  | Yes |  | Not Applicable |
| --- | --- | --- | --- | --- | --- |

| Author/Year | Criteria 1 | Criteria 2 | Criteria 3 | Criteria 4 | Criteria 5 | Criteria 6 | Criteria 7 | Criteria 8 | Criteria 9 | Criteria 10 |
| --- | --- | --- | --- | --- | --- | --- | --- | --- | --- | --- |
| Botha et al., 2012 |  |  |  |  |  |  |  |  |  |  |
| Esfandiari et al 2019 |  |  |  |  |  |  |  |  |  |  |
| Fenton Et al 2016 |  |  |  |  |  |  |  |  |  |  |
| Le et al.  2018 |  |  |  |  |  |  |  |  |  |  |
| Sajid et al. 2021 |  |  |  |  |  |  |  |  |  |  |

Hoy et al’s Risk of Bias tool for quantitative studies. Criteria 1: Was the study’s target population a close representation of the national population in relation to relevant variables? Criteria 2: Was the sampling frame a true or close representation of the target population? Criteria 3: Was some form of random selection used to select the sample OR was a census taken? Criteria 4: Was the likelihood of nonresponse bias minimal? Criteria 5: Were data collected directly from the subjects? Criteria 6: Was an acceptable case definition used in the study? Criteria 7: Was the study instrument that measured the parameter of interest shown to have validity and reliability? Criteria 8: Was the same mode of data collection used for all subjects? Criteria 9: Was the length of the shortest prevalence period for the parameter of interest appropriate? Criteria 10: Were the numerator(s) and denominator(s) for the parameter of interest appropriate?
